# Sustainable performance evaluation of pharmaceutical companies: sustainable balanced scorecard and hybrid MCDM approach

**DOI:** 10.3389/fpubh.2024.1495156

**Published:** 2025-01-08

**Authors:** Deqiang Deng, Jiayang Zhang, Jingyi Wang, Xiuran Zong

**Affiliations:** ^1^College of Economics and Management, Nanjing Forestry University, Nanjing, Jiangsu, China; ^2^College of Economics and Management, Nanjing University of Science and Technology, Nanjing, Jiangsu, China

**Keywords:** sustainability performance, pharmaceutical company, sustainability balanced scorecard, multiple criteria decision making, analytical network process

## Abstract

Despite the increasing demand for sustainable development of pharmaceutical companies due to the rigorous pressure of environmental regulation, public health crisis and economic competition, there has been little research on relevant evaluation models. The COVID-19 experience has also prompted investors in pharmaceutical companies to re-examine the impact of environment and ethics on business development. Therefore, pharmaceutical companies need to focus on their performance, especially on the shift from a single financial performance to an integrated performance. This paper constructs a reticulated sustainable performance evaluation model for decision-makers based on the Sustainability Balanced Scorecard (SBSC) framework. The evaluation results are derived using Decision Making Experiment and Evaluation Laboratory (DEMATEL), Analytical Network Process (ANP) and modified VlseKriterijumska Optimizacija I Kompromisno Resenje (VIKOR). The model can help management gain a more comprehensive understanding of the company’s overall situation, promote management’s focus on the balance and synergies between the various dimensions and indicators of sustainability performance, clarify the relationships and the comment weights of evaluation dimensions and indicators, and provide sustainability improvement solutions, which have been neglected in previous research on the evaluation of sustainability performance of pharmaceutical companies. Based on questionnaires with experts, this paper finds that the Environment is the most important factor, followed by Internal Processes, Customers, Finance, Learning and Growth, as well as Society. The empirical results of a Chinese pharmaceutical company suggest that green transformation and customer relations are the priorities, in addition to the need for additional ways to improve the sustainability performance of pharmaceutical companies. The evaluation results provide a strategic reference for stakeholders, which helps the case company to find better strategies for sustainable development and priorities for improving their sustainability performance.

## 1 Introduction

As the second largest pharmaceutical market in the world, China’s pharmaceutical companies have reached a market size of $1.87 trillion by 2022. The market is growing with the aging of the global population (1), increasing health literacy, and rapid advances in biomedical technology (2, 3). Pharmaceutical companies inevitably generate a large amount of waste during the Research and Development (R&D) and production process, which poses a risk of environmental pollution (4, 5). Some studies say the pharmaceutical industry emits 1.5 times more CO2 than the automotive industry (6). With stricter emission requirements and the need for increasing social responsibility, it is important for pharmaceutical companies to achieve sustainable development. According to the Triple Bottom Line theory, pharmaceutical companies need to improve economic, environmental and social performance integratively when they pursue their sustainability performance. With regard to the economic aspect, the European Federation of Pharmaceutical Industries and Associations (EFPIA) has noted that pharmaceutical companies have been one of the most relevant sectors of the global economy. Concerning the environmental aspect, pharmaceutical companies are not environmentally friendly because the pharmaceutical industry generates a series of environmental pollution during production (7, 8). In terms of the social aspect, pharmaceutical companies are generally perceived to have greater responsibilities and challenges concerning social issues, especially during public health crises (e.g., the COVID-19 pandemic) (9, 10). Although taking on more social responsibility can be less economically profitable in the short term for pharmaceutical companies, it can reap a better reputation and build a good corporate image in the long run. Therefore, it is strategically crucial for the pharmaceutical industry to focus on economic, environmental and social sustainability performance (11, 12).

Sustainability is essential for business success, especially for pharmaceutical companies. Putting sustainability at the heart of a company’s strategic objectives can help companies achieve better long-term benefits (13). Investors are also beginning to focus on sustainability criteria when evaluating company performance (11), and customers are more inclined to choose more sustainable products (14). Employee rights and working environments face greater societal scrutiny (15). The pursuit of balance and coordination among economic performance, social performance and environmental performance is the inevitable trend of sustainable development of enterprise organizations. Therefore, it is necessary and meaningful to establish a sound methodology for evaluating the sustainability performance of pharmaceutical companies. An excellent sustainability performance evaluation model can help the management of pharmaceutical companies answer three questions: (1) What is the conceptual framework (including the dimensions and indicators) for sustainability performance evaluation of pharmaceutical companies considering the balance and synergy between economic performance, social performance and environmental performance? (2) How to determine the weights considering the network relationship between the dimensions and indicators of sustainability performance evaluation of pharmaceutical companies? (3) How to measure sustainability performance and provide actionable recommendations and guidelines for improving the sustainability performance of pharmaceutical companies?

Unfortunately, prior literature on pharmaceutical companies did not answer well the above questions. Firstly, in terms of the conceptual framework for evaluating the sustainability performance of pharmaceutical firms, many studies have focussed on the profitability of pharmaceutical firms (16, 17). Related scholars have also used company share prices to measure the financial health of pharmaceutical companies (18). With the increasing awareness of social responsibility and environmental protection, Kim and Lee (19) confirmed that sustainable development of pharmaceutical companies needs to focus on social, economic and environmental performance. Some studies are based on triple bottom line theory [e.g., (20, 21)], which focuses on economic, environmental and social dimensions. However, the balance and synergies between these three conceptual dimensions have not received attention. For assessing the sustainable performance evaluation of pharmaceutical companies cannot focus on one or several dimensions independently, but should focus on the balance and flexibility between them. The Sustainability Balanced Scorecard (SBSC) can help companies implement sustainability strategies, facilitate sustainability management decision-making and reporting, support regulatory data requirements, and meet stakeholder information needs on sustainability issues (22, 23). Therefore, this paper applies the SBSC as a conceptual framework for evaluating the sustainability performance of pharmaceutical companies from a multidimensional perspective.

Secondly, in terms of the relationship between dimensions and indicators for evaluating the sustainability performance of pharmaceutical companies, although some studies have begun to emphasize the integration of environmental, social and economic dimensions, they have neglected that the three dimensions of sustainability are not independent of each other. For example, the increasing financial profit of an enterprise will provide more financial support to carry out environmental protection. Meanwhile, environmental protection work will also bring a better reputation to the company, which can boost the financial profits of the company in the long run (24). The United Nations Department of Economic and Social Affairs (UN DESA) has recommended the need to establish interlinkages between the social, economic and environmental aspects of sustainable development. This paper adopts a non-hierarchical network-like SBSC and can be fully integrated with the TBL theory. Previous literature has mostly used hierarchical and semi-equivalent mechanisms of SBSC, which do not well reflect the synergies between the various evaluation indicators of sustainable development of pharmaceutical companies. Furthermore, this paper used the Decision Making Experiment and Evaluation Laboratory (DEMATEL) method to analyze the network relationship between dimensions and indicators for evaluating the sustainability performance of pharmaceutical companies and the Analytical Network Process (ANP) method based on DEMATEL is also used to determine the weights between dimensions and indicators.

Finally, in terms of measuring the sustainability performance of pharmaceutical companies, previous studies have primarily used Data Envelopment Analysis (DEA) methods (21, 25–27). Traditional DEA models for measuring the efficiency of decision-making units (DMU) require that inputs and outputs be positive, quantitative and precise. However, in many real-life problems, the data are expressed as qualitative, imprecise and ambiguous. As a result, DEA results are not valuable for the multicriteria decision model (28). Therefore, this paper uses the modified VlseKriterijumska Optimizacija I Kompromisno Resenje (VIKOR) method to address this issue and identify the performance gap for future development. The modified VIKOR method can find the closest compromise to the optimal solution (28) and has been successfully applied to many practical questions of decision-making (29, 30).

Compared to previous sustainability performance evaluation models for pharmaceutical companies, our model provides more valuable alternatives for decision-makers of pharmaceutical companies. Firstly, this model considers multiple balanced performance dimensions simultaneously based on the framework of SBSC, which can provide more useful information for decision makers to construct a reticulated sustainable performance evaluation model. Secondly, some statistics and economics are unrealistic in reality because they ignore the links between the dimensions and indicators of sustainability performance evaluation (31, 32). Therefore, our model can provide a more balanced and comprehensive sustainability performance evaluation for pharmaceutical companies. Thirdly, this model not only evaluates the holistic sustainability performance but also finds alternatives for future improvements, which can help the management of pharmaceutical companies to enhance sustainable competitive advantage.

This paper is structured as follows. The following section reviews the current literature on the sustainability performance of pharmaceutical companies, SBSC and its application. Section 3 shows the research methodology, which proposes a sustainability performance evaluation model for pharmaceutical companies based on the SBSC, DEMATEL with ANP and modified VIKOR methods. Then, Section 4 presents a case study of a Chinese pharmaceutical company using this sustainability performance evaluation model, including some management implications and discussions for improving its sustainability performance. Finally, Section 5 summarizes the contributions, recommendations, limitations, and further research.

## 2 Literature review

### 2.1 Development and application of performance evaluation tools

As one of the most widely used performance assessment tools, Objectives and Key Results (OKR) is a corporate management tool that evaluates the weighting of the importance of each outcome by examining the deployment and implementation of strategies (33, 34). However, due to its difficulty and operational burden, the use of OKR is much less effective if it fails to cope with numerous problems. Another performance evaluation model is Balanced Scorecard (BSC), which is known for its comprehensive and balanced multidimensional performance measurement model that considers both financial and non-financial aspects. However, the BSC still has limitations because it does not take into account social and environmental objectives (35). Kaplan and Norton (36) highlight that some organizations may require additional performance perspectives beyond the four in the original BSC. There-fore, it is not controversial to add more performance perspectives to traditional BSC for addressing environmental and social goals (37). According to the Triple Bottom Line theory, companies should pay attention to both social and environmental performance besides economic performance. Many industries, including the pharmaceutical industry, are challenged to consider social and environmental standards in response to sustainability requirements from a variety of stakeholders, including regulators, media, customers, and supply chain partners, etc. (38). Therefore, developing an extended BSC that takes economic, social, and environmental performance is necessary to assess a company’s sustainability. That is, SBSC is derived from the traditional BSC to provide decision-makers with more valuable sustainability information, including economic, environmental and social sustainability (39).

The SBSC can be used as a multi-dimensional performance measurement and management control tool to play an important role in the sustainable development of enterprises. Numerous studies have shown that SBSC can address a range of management needs related to corporate sustainability issues, namely assisting companies in implementing sustainability strategies, promoting sustainability management standards and decisions, supporting regulatory data requirements, and meeting the information needs of stakeholders (23). As to the structure of SBSC, some scholars call for strict hierarchical causality (40), others propose semi-hierarchical architectures (41), and others propose network-like architectures (42). Therefore, SBSC is a suitable tool to evaluate sustainability performance because it can provide more accurate and valuable information.

### 2.2 Sustainability performance of pharmaceutical companies

Sustainable development is necessary to meet current needs without compromising the ability of future generations to meet their needs, while protecting the Earth’s ecosystems and life-supporting capacity (43). Pharmaceutical companies are expected to be leaders in sustainable development (44). Over the last two decades, sustainability has entered the realm of importance for pharmaceutical companies (45). Many studies have highlighted the sustainability issues in the pharmaceutical sector (46). Schneider et al. (46) analyzed the evolution of reported sustainability activities in the pharmaceutical industry, which showed an increase in the range and extent of relevant activities. They found that the number and form of pharmaceutical companies’ sustainability-related activities varied considerably between companies worldwide. They suggested that pharmaceutical companies need to develop strategies to address the new sustainability challenges.

Some studies focus on the particular dimension of the sustainability performance of pharmaceutical companies. For example, SubbaNarasimha et al. (21) used DEA to evaluate the performance of 29 US pharmaceutical companies based on mainly investigating the economic performance indicators. Moslemi et al. (25) identified a conceptual framework for assessing the economic and social performance of a pharmaceutical company’s supply chain based on a revised balanced scorecard. Bhattacharyya and Chatterjee (26)used DEA to measure the financial efficiency of selected Indian pharmaceutical companies under the product patent system. Joshi et al. (47) used the fuzzy Delphi method and analytic hierarchy process to study the environmental performance of the green supply chain of pharmaceutical companies to achieve sustainability. With growing interest in corporate sustainability performance management and measurement systems, numerous stakeholders, including ceos and consumers, have emphasized the importance of assessing the sustainability of their companies (48). Therefore, some studies began to emphasize the integration of environmental, social and economic dimensions of sustainability performance [e.g., (27)].

### 2.3 A discussion about the prior literature

Firstly, while there is widespread interest in assessing the sustainability performance of pharmaceutical companies, some studies focus on one or more economic, environmental, and social aspects without looking at the balance and flexibility between them. The findings of Dkhili (49) showed that environmental performance is positively related to the financial profitability of the firm. Xu et al. (50) also showed that firms that perform well in Corporate Social Responsibility (CSR) have better profitability than firms that perform poorly. Based on the traditional BSC, the SBSC explicitly incorporates strategically relevant environmental, social and ethical objectives (51). Therefore, this paper adds social and environmental dimensions to the traditional BSC model to construct a six-dimensional Sustainability Balanced Scorecard for evaluating the sustainability performance of pharmaceutical companies.

Secondly, for a better understanding of the company’s sustainability shortcomings and targeted improvement, it is required to consider the social, environmental and economic dimensions in a holistic manner (52). Therefore, the linkages between social, environmental and economic dimensions cannot be ignored. For example, the implementation of ISO 14001 certification has a positive influence on cleaner production, which is also conducive to economic sustainability and social sustainability (53). The DEMATEL method allows for a structural model involving causal relationships, which can effectively explore the links between pharmaceutical company evaluation dimensions and criteria (54–56). This paper uses the combination of DEMATEL and ANP methods that can obtain common weights for real-world interdependent dimensions and indicators, effectively providing a network analysis of the sustainability performance evaluation dimensions and indicators of pharmaceutical companies.

Thirdly, some studies use the DEA approach to assess the sustainability performance of pharmaceutical companies [e.g., (21, 27)]. Given the unavailability of precise quantitative data, traditional DEA models do not quasi-accurately yield results in Multiple criteria decision making (MCDM) (28). Similarly, while PLS-SEM is highly favorable for researchers relying on relatively small samples and non-normal data (57), it also enables researchers to deal with both reflective and formal measures (58). However, when applying PLS-SEM, researchers need to perform various robustness checks before and after estimation to avoid some non-normality or unobserved situations that affect the validity of the results. The modified VIKOR can assess and identify performance gaps for future development, which further provides a reasonable and valid evaluation of the sustainability performance of pharmaceutical companies (29).

Finally, few studies have used the SBSC-based MCDM approach to empirically assess the sustainability performance of pharmaceutical companies. Therefore, this paper combines DEMATEL, ANP, and improved SBSC-based VIKOR to build a hybrid MCDM model for evaluating the sustainability performance of pharmaceutical companies, and applies it to a case study of Chinese pharmaceutical companies.

## 3 Methods

### 3.1 Evaluation framework for sustainability performance of pharmaceutical companies based on SBSC

**Figure 1:**
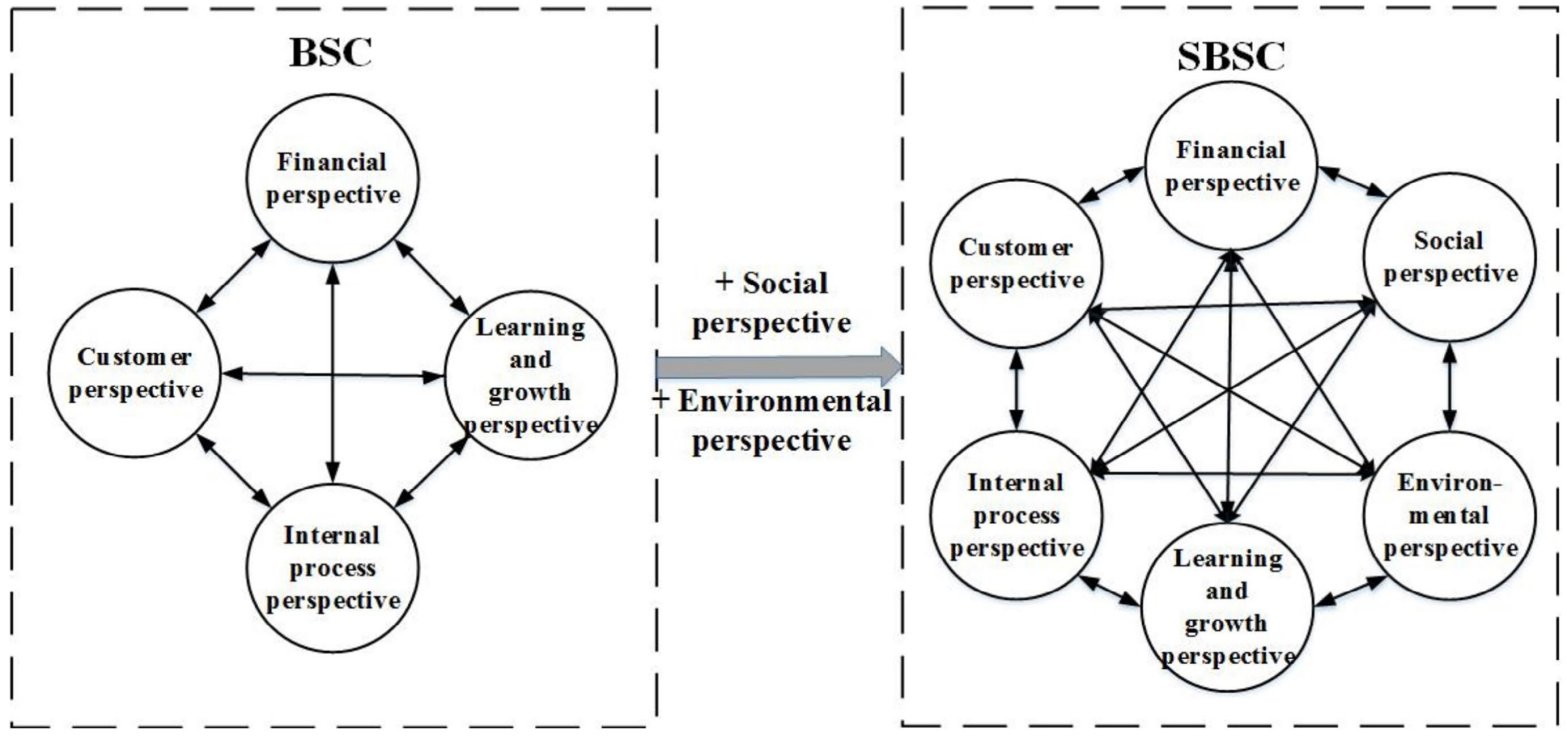
SBSC framework for evaluating the Sustainable performance of pharmaceutical companies.

BSC has been increasingly linked to strategic planning and implementation as a management framework to help identify key value drivers (59). However, BSC is unsuitable for measuring sustainability performance (60). According to the prior studies, to provide a comprehensive and effective response to the different dimensions of sustainability in line with the Triple Bottom Line theory, this paper constructs the SBSC framework by combining the traditional four dimensions of the BSC framework with environmental and social dimensions, as shown in Figure 1.

### 3.2 Evaluation indicators for the sustainability performance of pharmaceutical companies

This paper uses a questionnaire to identify indicators to evaluate the sustainability performance of pharmaceutical companies from a pool of indicators including 23 evaluation indicators based on the previous literature. The interviewees are some experts in the area of sustainability performance management. The questionnaire is scored on a five-point Likert scale in which respondents rate the extent of the importance of each evaluation indicator (from 1 to 5: very unimportant, unimportant, average, important, very important). 63 questionnaires were distributed, and 30 questionnaires were returned. In the literature that uses similar evaluation methods [e.g., (61–63)], the number of valid questionnaires recovered was 9, 8 and 10, and the samples were less than the valid samples retrieved in this paper. Therefore, the data analysis based on the number of samples recovered in this paper is still highly persuasive. Table 1 provides the descriptive information of the interviewees.

**Table 1:** Detailed information about the interviewees for identifying performance indicators.

Category	Content	Number	Percentage
Gender	Male	14	47%
	Female	16	53%
Age	Under 30 years old	8	27%
	30 ~ 40 years old	17	56%
	40 ~ 50 years old	2	7%
	50 years old and above	3	10%
Status	Academicians who study the sustainability performance	15	50%
	Top management who work on the sustainability performance	15	50%
Years of experience in research/practice on the sustainability performance	Less than 5 years	2	7%
	5 to 10 years	7	23%
	10 to 15 years	9	30%
	15 years or more	12	40%
Education	PhD	3	10%
	Master	6	20%
	Bachelor	21	70%

Finally, 12 indicators with an average score of 4 (important) and above were retained. The evaluation indicators for the sustainability performance of pharmaceutical companies are shown in Table 2.

**Table 2:** The evaluation indicator for sustainable performance of pharmaceutical companies.

SBSC Dimensions	Indicator	Description	Documentary sources
Financial Perspective (D1)	Sales Margin (C11)	the profitability of a business based on sales revenue	Wu (72) and Tavana (27)
	Sales Growth Rate (C12)	the ratio of the growth in sales revenue for analyzing the growth status and development capacity	Wu (72) and SubbaNarasimha (21)
Customer Perspective (D2)	Customer Satisfaction (C21)	the level of customer satisfaction with the company’s products and services to provide insight into the specific needs of customers for sustainable development	Mani et al. (73), Tavana (27), and Wu (72)
	Customer Relationship (C22)	the relationship that a company proactively establishes with its customers to achieve its business objectives	Sahu and Kohli (74), Chaar et al. (75), and Creamer and Freund (76)
Internal Process Perspective (D3)	Product Quality and Innovation Capacity (C31)	the level of the company’s product quality	Tavana (27), Eccles et al. (77), and Maltz et al. (78)
	Operational and Innovation Resource Input (C32)	the level of the company’s operational and innovation resource	Mani et al. (73) and Lorenzini et al. (79)
Learning and Growth Perspective (D4)	Employee Satisfaction (C41)	the level of employee satisfaction with salary, working environment and the development status of the company	Mani et al. (73) and Veleva et al. (80)
	Employee Training (C42)	the level of training and education to enhance the employee’s core competencies and capabilities	Mani et al. (73), Tavana (27), Saeed et al. (81), and Bom et al. (82)
Social Perspective (D5)	Corporate Social Image (C51)	the image of a company in the minds of relevant stakeholders	Mani et al. (73), Tavana (27), and Agrawal et al. (83)
	Corporate Social Contribution (C52)	the extent to which a company contributes to society, including high-quality products and services, bearing appropriate taxes, solving employment problems, etc	Mani et al. (73) and Wu (72)
Environmental Perspective (D6)	Pollutant and Waste Treatment Capacity (C61)	the company’s ability to reduce the pollution level of pollutants and recycle waste.	Mani et al. (73), Tavana (27), and Veleva et al. (80)
	Investment in Environmental Resources (C62)	the company’s investment in environmental protection	Mani et al. (73), Sudhakar et al. (84), Ángel del Brío et al. (85), and Jabbour (86)

### 3.3 Evaluation methods for sustainability performance of pharmaceutical companies based on hybrid MCDM approaches

#### 3.3.1 Building an influence network relationship map based on DEMATEL

The DEMATEL approach allows for constructing an influence network relationship map (INRM) to show the relationships between these evaluation dimensions and indicators. The DEMATEL method can explain specific societal issues based on the network relationship maps and structural models. These basic concepts were used to create a series of new hybrid MCDM models, which can effectively solve complex and dynamic real-world problems (54, 55, 64–66). The detailed steps of the DEMATEL method are as follows.

*Step 1*: Construction of average score matrix

Calculate a score-based direct influence-relation average matrix F. Assume that there is P number of experts in the study of sustainability performance in pharmaceutical companies and n indicators. Each expert was asked to compare pairwise any two indicators with the integer score from 0 to 4, expressing the range from “absolutely no influence (0)” to “very high influence (4).” $x_{ij}$ shows the influence degree that indicator i affects indicator j. The questionnaire for each expert forms an n×n non-negative matrix $X^{p}$ = ${\left[x_{ij}^{p}\right]}_{n\times n}$, *p* = 1, 2, …, P. Where $X^{1}$,…,$X^{p}$,…,$X^{P}$ are the response matrices by P number of experts, and the elements of $X^{P}$ are denoted by $x_{ij}^{p}$ from domain expert p. Thus, an expert’s n×n average matrix F can be created by the Equation 1:


(1) $$F=\left[\begin{array}{cccc} f_{11} & f_{12} & \cdots & f_{1n}\\ f_{21} & f_{22} & \cdots & f_{2n}\\ \vdots & \vdots & \ddots & \vdots \\ f_{n1} & f_{n2} & \cdots & f_{nn} \end{array}\right]$$

The average scores of the X domain are $f_{ij}=\frac{1}{P}\sum_{f=1}^{P}x_{ij}^{p}$. This average matrix is the “initial direct relationship matrix Z” and represents the degree of influence of one indicator on other indicators, as well as the degree of influence from other indicators.

*Step 2*: Normalizing the initial direct influence relation average matrix

The normalized initial direct influence relation matrix D can be obtained by normalizing the average matrix F. The matrix Y can be derived from Equations 2, 3, and all principal diagonal criteria values are equal to zero:


(2) Y=s·F

(3) $$s=\min\left\{\frac{1}{\max_{1\leq i\leq n}\sum_{j=1}^{n}f_{ij}},\frac{1}{\max_{1\leq j\leq n}\sum_{j=1}^{n}f_{ij}}\right\}$$

*Step 3*: Constructing the total influence-relation matrix

A continuous decrease of the indirect effects of problems moves with the powers of the matrix Y, e.g., $Y^{2}$,$Y^{3}$,…,$Y^{\infty }$, and $\lim_{k\to \infty }Y^{k}$ = ${\left[0\right]}_{n\times n}$, for $\lim_{k\to \infty }$(I+Y+$Y^{2}$+…+$Y^{k}$)=${\left(I-Y\right)}^{-1}$, Where I is the unit matrix of n×n. The influence-relation matrix T is a n×n unit matrix, as shown in Equation 4.


(4) $$\begin{array}{c} \;\\ T=Y+Y^{2}+\ldots +Y^{K.}\\ \begin{array}{c} =Y\left(I+Y+\ldots +Y^{k-1}\right)\\ =Y\left(I+Y+\ldots +Y^{k-1}\right)\left(I-Y\right){\left(I-Y\right)}^{-1}\\ =Y{\left(I-Y\right)}^{-1},\text{where}\;\lim_{k\to \infty }Y^{k}={\left[0\right]}_{n\times n}\; \end{array} \end{array}$$

The total influence-relation matrix t for INRM can be derived from the above Equation 4, and the following Equations 5, 6 are used to generate the sums of the columns and rows of matrix t, respectively.


(5) $$\begin{array}{c} d={\left(d_{i}\right)}_{n\times 1}={\left[\sum_{j=1}^{n}t_{ij}\right]}_{n\times 1}\\ ={\left(d_{1},\ldots ,d_{2},\ldots ,d_{n}\right)}^{\prime } \end{array}$$

(6) $$\begin{array}{c} r={\left(r_{j}\right)}_{n\times 1}={\left(r_{j}\right)}_{1\times n}^{\prime }={\left[\sum_{j=1}^{n}t_{ij}\right]}_{1\times n}^{\prime }\\ ={\left(r_{1},\ldots ,r_{2},\ldots ,r_{n}\right)}^{\prime } \end{array}$$

Where $d_{i}$ is the sum of a row in the total influence relation matrix T, and represents the total influence of indicator/dimension i on all other indicators/dimensions [∑j=1ntij]An×1. And $r_{j}$ is the column sum in the total influence relation matrix T, and represents the total influence of indicator/dimension j received from all other indicators/dimensions ${\left[\sum_{j=1}^{n}t_{ij}\right]}_{1\times n}^{\prime }$. Thus, when i=j,$d_{i}-r_{i}$ offers an index of the strength of the total influences given and received, that is $d_{i}+r_{i}$ indicating the important degree of the indicator/dimension i plays in the system. In addition, $d_{i}-r_{i}$ provides an index of the degree of the cause of total influence. If $d_{i}-r_{i}$ positive, then indicator/dimension i is a net influencer, and if $d_{i}-r_{i}$ is negative, then indicator/dimension i is a net influenced.

#### 3.3.2 Determining the weights by DANP

In 1996, Saaty proposed ANP which forms the relationships in a model and enables a systematic portrayal of decision problems. Using ANP to find the weights of dimensions or indicators based on DEMATEL can provide a structure for decision-makers’ preferences and more effectively address the interactions between dimensions or indicators. Therefore, this paper combines the strengths of ANP and DEMATEL to address the issues of interdependence, feedback between indicators, and comment sets of weights (67–71). The specific steps are as follows:

*Step 4*: Construct the total influence relation matrix

The DEMATEL is used to derive the total influence relation matrix T from each dimension, with different degrees of influence relation for the indicators. The total influence relation matrix T of the primary indicators is summed over the rows of the indicators to obtain the row sums, which are then divided by the corresponding row sums for each primary indicator. The secondary indicators are normalized in each sub-matrix by summing the rows of the total impact matrix $T_{C}$ and dividing each indicator by its corresponding row sum, so that each sub-matrix has a row sum of 1 because of the normalization, as is shown in Equation 7.


(7) TC=D1AA⋮AADiAA⋮AAADnc11c12⋮c1m1Aci1ci2⋮cimiA⋮Acn1cn2⋮cnmnTC11⋯TC1j⋯TC1n⋮A⋮A⋮TCi1⋯TCij⋯TCin⋮A⋮⋱⋮TCn1⋯TCnj⋯TCnnc11⋯c1m1⋯ci1⋯cimi⋯cn1⋯cnmnD1⋯DiA⋯AADnA

Where $D_{n}$ is the nth cluster; $c_{\text{nm}}$ is the mth indicator in the nth dimension; and $T_{C}^{ij}$ is a submatrix of the influence relation by the indicators from a comparison of the ith dimension and the jth dimension. In addition, if the ith dimension has no influence on the jth dimension, then submatrix $T_{C}^{ij}=\left[0\right]$, shows independence (no influence relation) in each indicator on other indicators.

*Step 5*: Form an unweighted supermatrix w

Normalize the total influence relation matrix $T_{C}$ as shown in Equation 8:


(8) TCα=D1AA⋮AADiAA⋮AAADnc11c12⋮c1m1⋮ci1ci2⋮cimi⋮cn1cn2⋮cnmnTCα11⋯TCα1j⋯TCα1n⋮A⋮A⋮TCαi1⋯TCαij⋯TCαin⋮A⋮A⋮TCαn1⋯TCαnj⋯TCαnnc11⋯c1m1cj1⋯cjmj⋯cn1⋯cnmnD1ADjAAAADnA

Where $T_{C}^{\alpha }$ represents the normalizing total influence relation matrix, and $T_{c}^{\alpha 12}$ can be derived from Equations 9, 10; and $T_{c}^{\alpha nn}$ can be similarly obtained.


(9) $$T_{i}^{12}=\sum_{j=1}^{m_{2}}T_{ij}^{12},i=1,2,\cdots ,m_{1}$$

(10) Tcα12=AT1112/T112⋯T1j12/T112⋯T1m212/T112⋮A⋮A⋮Ti112/Ti12⋯Tij12/Ti12⋯Tim212/Ti12⋮A⋮A⋮Tm1112/Tm112⋯Tm1j12/Tm112⋯Tm1m212/Tm112=T11α12⋯T1jα12⋯T1m2α12⋮A⋮A⋮Ti1α12⋯Tijα12⋯Tim2α12⋮A⋮A⋮Tm11α12⋯Tm1jα12⋯Tm1m2α12

Based on the pairwise comparisons within the criteria, and the basic concept of ANP, the unweighted supermatrix W can be constructed by transposing the normalized influence-relation matrix by dimensions (or cluster), i.e., $W={\left(T_{C}^{\alpha }\right)}^{\prime }$, as shown in Equation 11.


(11) W=TCα′=D1AA⋮AADjAA⋮AAADnc11c12⋮c1m1⋮cj1cj2⋮cjmj⋮cn1cn2⋮cnmnW11⋯Wi1⋯Wn1⋮A⋮A⋮W1j⋯Wij⋯Wnj⋮A⋮A⋮W1n⋯Win⋯Wnnc11⋯c1m1…ci1⋯cimi⋯cn1⋯cnmnD1ADiAAAADnA

*Step 6*: Derived the weighted supermatrix $W^{\alpha }$. The total influence-relation matrix $T_{D}$ of dimensions is obtained according to the DEMATEL method, as given by Equation 12:


(12) TD=TD11⋯TD1j⋯TD1n⋮A⋮A⋮TDi1⋯TDij⋯TDin⋮A⋮A⋮TDn1⋯TDnj⋯TDnn

The normalized total influence-relation matrix of dimensions can be derived from the total influence-relation matrix divided by, as shown in Equation 13.


(13) TDα=TD11/T1⋯TD1j/T1⋯TD1n/T1⋮A⋮A⋮TDi1/Ti⋯TDij/Ti⋯TDin/Ti⋮A⋮A⋮TDn1/Tn⋯TDnj/Tn⋯TDnm/Tn=TDα11⋯TDα1j⋯TDα1n⋮A⋮A⋮TDαi1⋯TDαij⋯TDαin⋮A⋮A⋮TDαn1⋯TDαnj⋯TDαnn

The normalized $T_{D}^{\alpha }$ and the unweighted supermatrix W (shown as Equation 11), and the weighted supermatrix $W^{\alpha }$ (normalized supermatrix) can be easily obtained by Equation 14


(14) Wα=TDα×W=TDα11×W11⋯TDαi1×Wi1⋯TDαn1×Wn1⋮A⋮A⋮TDα1j×W1j⋯TDαij×Wij⋯TDαnj×Wnj⋮A⋮A⋮TDα1n×W1n⋯TDαin×Win⋯TDαnn×Wnn

*Step 7*: Calculate the limit supermatrix $W^{\alpha }$. Limit the weighted supermatrix by raising it to the kth power, until the supermatrix has converged and become a stable supermatrix. The global priority vectors are derived, which are known as the DANP influential weights, such as $\lim_{h\to \infty }{\left(W^{\alpha }\right)}^{h}$, where h represents any number of power.

#### 3.3.3 Measuring the satisfactory performance by modified VIKOR

In order to solve the problem of decision making in the context of conflicting multidimensions, Opricovic and Tzeng (28) propose the VIKOR method. An ideal solution is first determined and the VIKOR method calculates the gap between the actual values and the ideal and negative ideal solutions respectively, based on the management’s evaluation of each indicator. The final ranking is based on the difference. The closer to the ideal solution the closer to the desired value, and conversely the closer to the negative ideal solution the further away from the desired value. Priority is given to improving the indicators with larger gaps. Based on the ranking results, effective improvement strategies are proposed for the sustainable performance of pharmaceutical companies. The steps for implementing the modified VIKOR method are as follows:

*Step 8*: Determine the positive-ideal solution and negative-ideal solution and replace aspiration levels and worst value to adapt the current world situation. The aspiration level of j indicator is defined as $f_{j}^{\text{aspire}}$ and the worst value $f_{j}^{\text{worst}}$ for all indicators, which can be derived from the traditional form to the modified form. Export the positive-ideal solution and negative-ideal solution from the traditional approach are as follows:

Positive-ideal solution: $f^{*}=\left(f_{1}^{*},\ldots ,f_{j}^{*},\ldots ,f_{n}^{*}\right)$, where $f_{j}^{*}=\max_{k}\left\{f_{kj}|,,,k=1|,,,2|,,,\ldots |,,,m\right\}$;

Negative-ideal solution: $f^{-}=\left(f_{1}^{-},\ldots ,f_{j}^{-},\ldots ,f_{n}^{-}\right)$, where $f_{j}^{-}=\min_{k}\left\{f_{kj}|,,,k=1|,,,2|,,,\ldots |,,,m\right\}$;

*Step 9*: The modified method for replacement by “aspiration level” and “worst value.”

Aspiration level: $f^{\text{aspire}}=\left(f_{1}^{\text{aspire}},\ldots ,f_{j}^{\text{aspire}},\ldots ,f_{n}^{\text{aspire}}\right)$, where $f_{j}^{\text{aspire}}$ is the best value;

Worst level: $f^{\text{worst}}=\left(f_{1}^{\text{worst}},\ldots ,f_{j}^{\text{worst}},\ldots ,f_{n}^{\text{worst}}\right)$, where $f_{j}^{\text{worst}}$ is the worst value.

In this research, the satisfaction performance scores from 1 to 10, with “10” set as the desired value.

*Step 10*: Determine the average group utility of the gap and develop a prioritized improvement strategy.

$f_{i}^{*}=\max_{j}f_{ij},f_{i}^{-}=\min_{j}f_{ij}$ Find the best and worst values of i = 1,2,3,…,n.

$S_{j}=$ $\sum_{i=1}^{n}w_{i}$(|$f_{i}^{*}-f_{ij}^{-}|$)/(|$f_{i}^{*}-f_{i}^{-}$|)

$R_{j}=$ $\max_{i}w_{i}\left(f_{i}^{*}-f_{ij}^{-}\right)/\left(f_{i}^{*}-f_{i}^{-}\right)$

Where $W_{i}$ is the weight of each indicator, indicating their relative importance.

Calculation of the composite index Q:


$$Q=v\left(S_{j}-S^{*}\right)/\left(S^{-}-S^{*}\right)+\left(1-v\right)\left(R_{j}-R^{*}\right)/\left(R^{-}-R^{*}\right)$$

**Figure 2:**
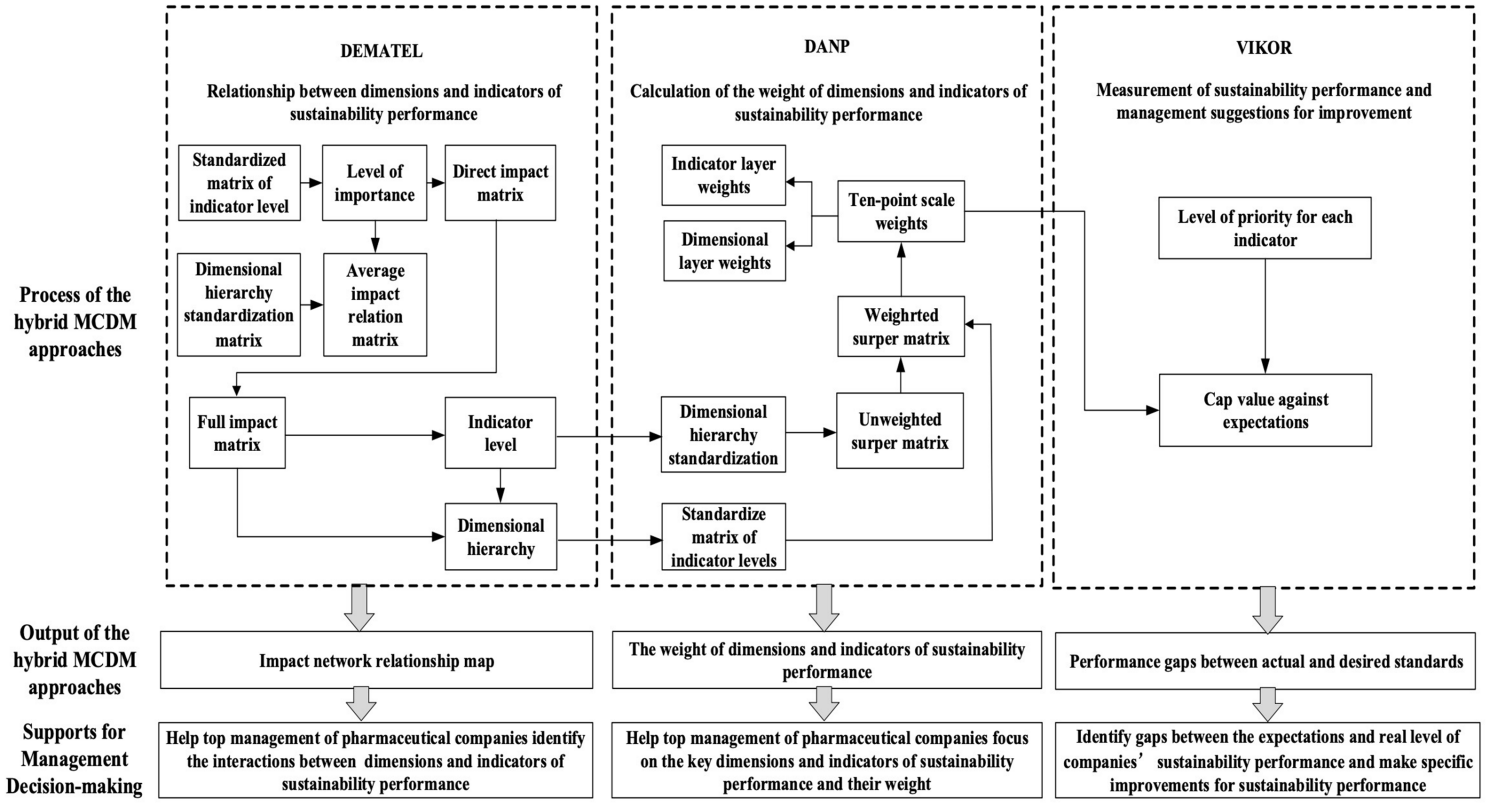
Flow chart of sustainability performance evaluation methods of pharmaceutical companies based on hybrid MCDM approaches.

Where $S^{*}=\min_{j}S_{j}$, $S^{-}=\max_{j}S_{j}$, $R^{*}=\min_{j}R_{j}$, $R^{-}=\max_{j}R_{j}$, j = 1,2,3…n, v as the maximum overall utility parameter, v = 0.5. Arrange all data in ascending order of Q, S and R. Q indicates the difference between the actual value and the ideal value; the smaller the Q, the closer the data is to the ideal value. The specific process of the sustainability performance evaluation methodology for pharmaceutical companies is shown in Figure 2.

## 4 Case study of a Chinese pharmaceutical company

### 4.1 Background and sustainability problem description of a Chinese pharmaceutical company

Nowadays, pharmaceutical companies in China are witnessing increasing sustainable development pressures. First, according to the data of Chinese pharmaceutical listed companies, under the multiple pressures of medical insurance negotiation, centralized procurement, innovation and R&D transformation, the profit space of pharmaceutical enterprises is shrinking. Second, because pharmaceutical enterprises are related to people’s livelihood and health, they are considered to bear greater social responsibility, which is more obvious during public health crises (e.g., the COVID-19 pandemic). Third, with the implementation of the Chinese National Emission Standard of Air Pollutants for Pharmaceutical Industry amended on 1st July 2019, pharmaceutical companies in China are facing more pressure from mandatory regulatory and economic penalties for illegal emissions and the environmental protection expectations of society.

AK Company is a typical pharmaceutical company that develops, produces and markets new drugs in East China. Facing the increasing sustainable development pressures, AK Company should pursue sustainability performance which is the synergy between economic performance, social performance and environmental performance. Accordingly, a sound model for evaluating the sustainability performance of AK Company is becoming essential for its business success. The model is expected to help the management of AK Company find the answers to its sustainability performance problem as follows: What is the interrelation between key factors of its sustainability performance assessment? How to evaluate the sustainability performance? What is the holistic level of sustainability performance? These answers can facilitate the management of AK Company to focus on the key points, develop the feasible strategies and improve the current weaknesses of sustainability performance.

### 4.2 Data collection

This paper adopts a two-stage questionnaire design, including expert questionnaire and senior management questionnaire. In the first stage, a questionnaire for experts was designed for evaluating the degree of influence between indicators of sustainability performance in pharmaceutical companies and determining the influential weight based on the DEMATEL and ANP methods. The questionnaire used a Likert scale to rate the degree of influence between indicators, with a scale of “0–4” indicating the degree of influence from lowest to highest. A total of 63 questionnaires were distributed and 50 results were validly returned. In the literature that uses similar evaluation methods [e.g., (61–63, 87)], the number of valid questionnaires recovered was less than 10. The interviewees were experts on sustainability performance management in pharmaceutical-related industries with at least five years of experience, which means those experts all had a profound understanding of the interrelationship between those evaluation indicators affecting sustainability performance. Therefore, the data analysis based on the number of samples recovered in this paper is still highly persuasive. Table 3 describes the details of the respondents who assessed the level of influence between sustainability performance indicators of pharmaceutical companies.

**Table 3:** Details of respondents used to assess the level of impact between indicators.

Category	Content	Number	Percentage
Gender	Male	24	48%
	Female	26	52%
Company Position	Management	7	14%
	Research & Development	8	16%
	Sales	7	14%
	Others	28	56%
Status	Academicians who study the sustainability performance	33	66%
	Top management who work on the sustainability performance	17	34%
Years of experience in research/practice on the sustainability performance	Less than 5 years	13	26%
	5 to 10 years	25	50%
	10 to 15 years	7	14%
	15 years or more	5	10%
Education	PhD	4	8%
	Master	22	44%
	Bachelor	18	36%
	Junior college	6	12%

In the second stage, a questionnaire for top management was developed to assess the satisfaction degree to evaluate AK Company’s sustainability performance by using the modified VIKOR method. The interviewees were core management within AK Company who had a comprehensive and accurate understanding of the actual performance level in the company. They gave their assessment of AK Company’s sustainability performance based on the performance indicators on a score of 0 to 10, with 0 being the very dissatisfaction (lowest score) and 10 being the very satisfaction (highest score). A total of 25 questionnaires were distributed and 23 were validly returned. The respondents of this questionnaire are only the management and core staff of the company, and the respondents are also required to have a certain level of understanding of sustainable development, so the questionnaire sample is small. However, this questionnaire involves almost all eligible respondents in AK Company, so it is sufficient to reflect the current status of the company’s sustainability. The descriptive analysis of the respondents of AK Company is shown in Table 4:

**Table 4:** Descriptive analysis of AK Company respondents.

Category	Content	Number	Percentage
Gender	Male	14	61%
	Female	9	39%
Age	Under 35 years old	5	22%
	35 ~ 45 years old	10	43%
	45 ~ 55 years old	8	35%
	55 years old and above	0	0%
Position	Management	9	40%
	Department Managers	4	17%
	Project Managers	4	17%
	Others	6	26%
Department	Production Department	3	13%
	R&D Department	3	13%
	Sales Department	4	17%
	Others	13	57%

### 4.3 Analysis of the relationship between dimensions and indicators based on the DEMATEL method

According to the step 1 of DEMATEL described above, an average initial direct-influence matrix shown in Table 5 was first obtained based on the data collected by interviews from the experts on the sustainability performance management in pharmaceutical-related industries. Then the normalization matrix shown in Table 6 was obtained by using the step 2 of DEMATEL. The total influence-relation matrix shown in Table 7 can be derived through Step 3 of DEMATEL on the basis of normalized direct influence relation matrix.

**Table 5:** Average initial direct-influence matrix.

	C11	C12	C21	C22	C31	C32	C41	C42	C51	C52	C61	C62
C11	0.00	3.04	2.66	3.00	3.24	2.40	2.88	2.72	2.70	2.96	2.04	2.30
C12	3.24	0.00	2.76	2.02	2.46	2.22	2.84	3.00	2.38	2.24	2.40	2.96
C21	3.34	2.22	0.00	2.20	2.88	1.98	2.72	2.32	2.72	3.34	1.60	1.82
C22	3.30	2.30	2.28	0.00	3.40	2.10	2.70	2.26	2.30	3.16	1.58	1.94
C31	3.12	2.96	1.86	3.12	0.00	2.80	2.16	2.58	2.74	2.80	1.70	2.04
C32	2.84	3.04	2.20	2.08	2.68	0.00	1.90	2.86	3.30	2.58	2.22	2.52
C41	2.20	1.98	2.16	2.48	2.26	2.72	0.00	1.96	2.12	2.80	1.92	1.90
C42	2.68	2.66	2.30	2.30	2.64	1.84	3.08	0.00	3.08	3.06	2.10	2.80
C51	3.06	2.26	2.66	3.24	3.16	2.46	2.62	2.48	0.00	2.28	2.14	2.20
C52	2.20	3.28	3.04	2.08	2.32	1.92	2.68	2.98	2.60	0.00	2.38	2.70
C61	2.08	2.90	2.98	2.26	1.96	2.12	1.88	2.60	2.22	2.76	0.00	3.10
C62	2.30	3.16	2.30	2.08	2.22	2.16	2.26	2.10	2.76	3.20	3.08	0.00

**Table 6:** Normalized direct influence relation matrix.

	C11	C12	C21	C22	C31	C32	C41	C42	C51	C52	C61	C62
C11	0.000	0.097	0.085	0.096	0.104	0.077	0.092	0.087	0.087	0.095	0.065	0.074
C12	0.104	0.000	0.089	0.065	0.079	0.071	0.091	0.096	0.076	0.072	0.077	0.095
C21	0.107	0.071	0.000	0.071	0.092	0.064	0.087	0.074	0.087	0.107	0.051	0.058
C22	0.106	0.074	0.073	0.000	0.109	0.067	0.087	0.072	0.074	0.101	0.051	0.062
C31	0.100	0.095	0.060	0.100	0.000	0.090	0.069	0.083	0.088	0.090	0.055	0.065
C32	0.091	0.097	0.071	0.067	0.086	0.000	0.061	0.092	0.106	0.083	0.071	0.081
C41	0.071	0.064	0.069	0.080	0.072	0.087	0.000	0.063	0.068	0.090	0.062	0.061
C42	0.086	0.085	0.074	0.074	0.085	0.059	0.099	0.000	0.099	0.098	0.067	0.090
C51	0.098	0.072	0.085	0.104	0.101	0.079	0.084	0.080	0.000	0.073	0.069	0.071
C52	0.071	0.105	0.097	0.067	0.074	0.062	0.086	0.096	0.083	0.000	0.076	0.087
C61	0.067	0.093	0.096	0.072	0.063	0.068	0.060	0.083	0.071	0.089	0.000	0.099
C62	0.074	0.101	0.074	0.067	0.071	0.069	0.072	0.067	0.089	0.103	0.099	0.000

**Table 7:** Total influence-relation matrix.

	C11	C12	C21	C22	C31	C32	C41	C42	C51	C52	C61	C62
C11	0.720	0.794	0.727	0.732	0.790	0.666	0.750	0.745	0.766	0.821	0.619	0.695
C12	0.782	0.675	0.702	0.677	0.738	0.635	0.720	0.723	0.727	0.770	0.605	0.686
C21	0.756	0.712	0.594	0.656	0.722	0.604	0.689	0.678	0.708	0.769	0.559	0.628
C22	0.759	0.719	0.665	0.593	0.740	0.611	0.692	0.680	0.700	0.768	0.561	0.635
C31	0.768	0.751	0.667	0.697	0.655	0.641	0.690	0.702	0.726	0.772	0.576	0.651
C32	0.768	0.761	0.684	0.675	0.741	0.565	0.690	0.717	0.749	0.774	0.598	0.671
C41	0.663	0.646	0.604	0.608	0.645	0.574	0.552	0.612	0.634	0.692	0.521	0.578
C42	0.767	0.753	0.689	0.685	0.743	0.624	0.726	0.635	0.746	0.791	0.597	0.682
C51	0.780	0.743	0.700	0.712	0.759	0.643	0.714	0.710	0.657	0.772	0.598	0.665
C52	0.746	0.762	0.703	0.670	0.726	0.619	0.708	0.715	0.725	0.694	0.598	0.672
C61	0.714	0.724	0.676	0.649	0.688	0.601	0.659	0.679	0.688	0.746	0.506	0.659
C62	0.736	0.748	0.673	0.660	0.711	0.616	0.684	0.681	0.718	0.775	0.609	0.583

**Figure 3:**
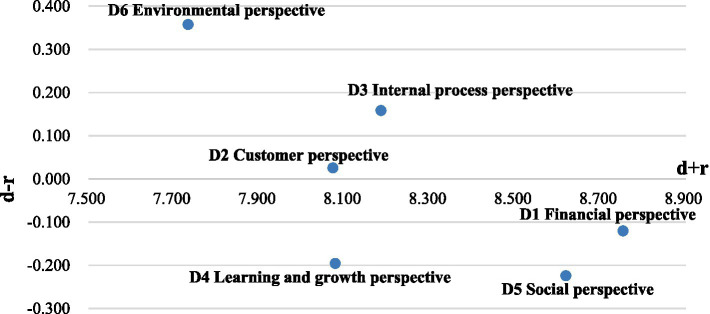
The influential network relations map for sustainable performance evaluation of pharmaceutical companies.

As revealed in Table 8, the sum of the effects of each dimension and metric can be derived by applying Equations 5, 6 in DEMATEL step 3. The INRM in Figure 3 illustrates the influential network relationship from SBSC six perspectives. The indicators with positive values of $d_{i}-r_{i}$ have a great influence on other indicators. The indicators with negative values of $d_{i}-r_{i}$ are greatly influenced by the other indicators. A significantly positive value of $d_{i}-r_{i}$ represents that this indicator affects other indicators much more than those other indicators affect it, which means it should be a priority for improvement.

**Table 8:** The sum of influences and ranking of each indicator.

Dimensions/Indicators	Row sum(di)	Column sum(ri)	(*d_i_*+*r_i_*)	(*d_i_*−*r_i_*)
D1 Financial Perspective	4.317	4.437	8.754	−0.120
C11 Sales Margin	1.515	1.502	3.017	0.012
C12 Sales Growth Rate	1.457	1.470	2.927	−0.012
D2 Customer Perspective	4.049	4.024	8.074	0.025
C21 Customer Satisfaction	1.250	1.259	2.509	−0.009
C22 Customer Relations	1.259	1.249	2.508	0.009
D3 Internal Process Perspective	4.172	4.014	8.186	0.158
C31 Product Quality and Innovation Capability	1.296	1.396	2.692	−0.100
C32 Operational and Innovation Resource Input	1.306	1.206	2.512	0.100
D4 Learning and Growth Perspective	3.942	4.137	8.079	−0.196
C41 Employee Satisfaction	1.163	1.278	2.441	−0.114
C42 Employee Training	1.361	1.247	2.608	0.114
D5 Social Perspective	4.198	4.422	8.620	−0.224
C51 Corporate Social Image	1.429	1.382	2.811	0.047
C52 Corporate Social Contribution	1.419	1.466	2.885	−0.047
D6 Environmental Perspective	4.046	3.688	7.734	0.357
C61 Pollutant and Waste Treatment Capacity	1.165	1.115	2.280	0.050
C62 Investment in Environmental Resources	1.192	1.242	2.434	−0.050

As shown in Table 8 and Figure 3, according to the value of influence given $d_{i}-r_{i}$, the following ranking of the importance of the dimensions affecting the evaluation of the sustainability performance of pharmaceutical companies was obtained: “D6: Environmental Perspective (0.357),” “D3: Internal Process Perspective (0.158),” “D2: Customer Perspective (0.025),” “D1: Financial Perspective (−0.120),” “D4: Learning and Growth Perspective (−0.196),” and “D5: Social Perspective (−0.224).” That is, “D6: Environmental Perspective,” “D3: Internal Process Perspective” and “D2: Customer Perspective” should be a priority for improvement. That is, in order to improve sustainability performance, pharmaceutical companies should firstly focus on environmental aspects, which requires not only investing in environmental protection but also improving their ability to deal with pollutants and waste. Furthermore, pharmaceutical companies need to increase their investment in research and development. By constantly updating their technology and innovation, they can gain broader customer satisfaction and relationship, which can supply a solid foundation to make more financial profits and social contributions.

### 4.4 Calculation of the weights of the indicators according to the DANP method

As shown in Table 9, the DANP impact weights for each substandard can be obtained by using a combination of DEMATEL and ANP methods. These weights were applied to the modified VIKOR method to assess the performance of each indicator.

**Table 9:** Influential weights for each indicator based on the DANP method.

Indicators	C11	C12	C21	C22	C31	C32	C41	C42	C51	C52	C61	C62
Weights	0.091	0.089	0.082	0.081	0.088	0.075	0.084	0.084	0.086	0.092	0.070	0.079

As can be seen from Table 9, in terms of the indicator level, corporate social contribution (C52) has the highest weight at 0.092, while pollutant and waste treatment capacity (C61) has the lowest at 0.070. It is crucial for pharmaceutical companies to pay attention to the environment and society besides the traditional BSC dimensions. Pharmaceutical companies not only need to increase their financial investment in environmental protection, but also to improve their environmental technologies, such as pollutant and waste treatment capabilities. It is also necessary to continuously improve social contributions to create a good image for the company and to raise social concerns.

### 4.5 Measuring sustainability performance according to the modified VIKOR method

The modified VIKOR method is applied to evaluate the overall performance gap of AK Company’s sustainability performance. Based on the weight of each indicator and dimension described above and the data from the core management’s assessment of AK Company’s sustainability performance, each indicator score and the total average gap ($S_{k}=\sum_{j=1}^{n}w_{j}r_{kj}$) are obtained by using the DANP weights. As shown in Table 10, in terms of dimensions, the gap values are ranked from largest to smallest: D2 (Customer Perspective) > D1 (Financial Perspective) > D5 (Social Perspective) > D4 (Learning and Growth Perspective) > D6 (Environmental Perspective) > D3 (Internal Process Perspective). In terms of indicators, the top five gaps are: C22 (Customer Relations) > C12 (Sales Growth Rate) > C62 (Investment in Environmental Resources) > C51 (Corporate Social Image) > C42 (Employee Training). AK company’s comprehensive average sustainability performance value is 6.631, which means a shortfall of approximately 33.7% gap from the expected aspiration level.

**Table 10:** Performance evaluation based on the VIKOR method.

	Local weight	Global weight (*by DANP*)	Level of performance	Relative gaps
D1 Financial Perspective	0.179		5.904	0.410
C11 Sales Margin	0.505	0.091	7.174	0.283
C12 Sales Growth Rate	0.495	0.089	4.609	0.539
D2 Customer Perspective	0.163		5.312	0.469
C21 Customer Satisfaction	0.502	0.082	7.087	0.291
C22 Customer Relations	0.498	0.081	3.522	0.648
D3 Internal Process Perspective	0.162		8.854	0.115
C31 Product Quality and Innovation Capability	0.539	0.088	9.957	0.004
C32 Operational and Innovation Resource Input	0.461	0.075	7.565	0.243
D4 Learning and Growth Perspective	0.167		6.652	0.335
C41 Employee Satisfaction	0.500	0.084	7.261	0.274
C42 Employee Training	0.500	0.084	6.043	0.396
D5 Social Perspective	0.179		6.163	0.384
C51 Corporate Social Image	0.483	0.086	5.826	0.417
C52 Corporate Social Contribution	0.517	0.092	6.478	0.352
D6 Environmental Perspective	0.149		7.063	0.294
C61 Pollutant and Waste Treatment Capacity	0.471	0.070	8.696	0.130
C62 Investment in Environmental Resources	0.529	0.079	5.609	0.439
Overall Performance Score			6.631	
Overall gap value				0.337

### 4.6 Management implications and discussion for the improvement of AK Company’s sustainability performance

According to the evaluation results, some management implications for the decision-maker of AK Company can be presented to improve its sustainability performance. In terms of key factors of pharmaceutical companies’ sustainability performance, the INRM as shown in Figure 3 states the order of priority for improving the pharmaceutical companies’ sustainability performance as follows: “D6: Environmental Perspective,” “D3: Internal Process Perspective,” “D2: Customer Perspective,” “D1: Financial Perspective,” “D4: Learning and Growth Perspective,” and “D5: Social Perspective.” This result supports AK Company should adopt the “Green Transformation Strategy.” A report by China’s Ministry of Environmental Protection showed that the total output value of the pharmaceutical industry accounted for less than 3% of the country’s GDP, while the total amount of pollution emissions accounted for 6%. As a typical pharmaceutical company, AK company is facing increasing pressure from environmental regulation, especially after the implementation of the Chinese National Emission Standard of Air Pollutants for Pharmaceutical Industry amended on 1st July 2019. Therefore, AK Company needs to increase investment in environmental protection and green innovation.

In terms of the gap in AK Company’s sustainability performance, the management should focus on five indicators: “Customer Relations,” “Sales Growth Rate,” “Investment in Environmental Resources,” “Corporate Social Image” and “Employee Training.” Firstly, because the customer is the object of production services of the enterprise, Customer Relations should be taken seriously. Handling customer relations well will help companies understand customer needs quickly and adjust their production and management goals better. Furthermore, better customer relations can improve the cooperation with customers, which can increase product sales and expand the company’s reputation. Therefore, AK Company should communicate with its customers promptly to understand their demands. Customers’ suggestions should be taken seriously and a return visit mechanism should be set up to facilitate the understanding of their shortcomings and correct them. Secondly, sales revenue is the main source of profit for the company and one of its main objectives. Therefore, AK Company should clarify its marketing strategy and develop proven methods to increase sales growth rates, which also provide financial support for other company activities including R&D. Thirdly, AK Company should consider the investment in environmental resources while dealing with pollutants. AK Company should focus on long-term benefits, adhere to a sustainable development strategy and put environmental protection into practice, especially the improvement of the environmental protection resource input system. At the same time, AK Company should strengthen environmental protection publicity and raise employees’ awareness of environmental protection. Environmental protection cannot be fully achieved by the efforts of management alone but requires the joint efforts of every employee. Fourthly, a good corporate social image can help the company gain public support and thus expand its sales. AK Company can actively fulfill its social responsibilities, enhance its corporate image and improve public understanding of the company. In addition, AK Company needs to improve the quality of its products, continue to innovate and strengthen cooperation, so as to fundamentally gain the public’s recognition and enhance public goodwill. Finally, AK Company should pay attention to the training of its employees. AK Company can arrange suitable jobs for each employee according to their strengths, so that they can maximize their value. Regular training should be organized for employees to improve their business level and motivate them to work.

## 5 Conclusion

As an industry related to human life and social welfare, pharmaceutical enterprises are facing the rigorous pressure of environmental regulation, public health crises (e.g., the COVID-19 pandemic) and economic competition. Pharmaceutical companies should establish proper sustainability performance evaluation models to strengthen competitive advantage and win public trust. Therefore, based on the SBSC framework, this study constructs a hybrid MCDM model combining DEMATEL with ANP and improved VIKOR to explore the sustainability performance evaluation of pharmaceutical companies.

This paper provides some contributions to the literature on the sustainability performance evaluation of pharmaceutical companies. Firstly, based on the Triple Bottom Line theory, this model extends the social and environmental dimensions into the traditional BSC framework to construct a six-dimensional Sustainability Balanced Scorecard for evaluating the sustainability performance of pharmaceutical companies. This SBSC framework can help management to focus on the balance and synergy between the six dimensions when implementing sustainability strategies. Secondly, this model using the DEMATEL approach can point out directions for improvement in the sustainability performance of pharmaceutical companies, rather than just performance rankings. The model helps stakeholders in the pharmaceutical industry understand the most important factors in the sustainability performance of the pharmaceutical companies through INRM, thus providing a holistic and comprehensive understanding of the network of influences between the various factors. Based on the data from experts, six dimensions of sustainability performance of pharmaceutical companies are prioritized from an SBSC perspective in the following order: “D6: Environmental Perspective,” “D3: Internal Process Perspective,” “D2: Customer Perspective,” “D1: Financial Perspective,” “D4: Learning and Growth Perspective,” and “D5: Social Perspective.” This means that Green Transformation should be an important strategic choice for pharmaceutical companies. Thirdly, this model based on the DANP approach can effectively address the relationship between the indicator for sustainability development performance of pharmaceutical companies. Therefore, the model provides pharmaceutical companies with effective and reasonable influential weights for the concrete implementation of sustainable performance evaluation in the real world. Finally, the modified VIKOR method is applied to determine an ideal solution and a negative ideal solution, and then the actual values are calculated based on management’s evaluation of each indicator in relation to the ideal and negative ideal solutions, respectively, and finally ranked according to the gap. The modified VIKOR method is effective in avoiding the “selection of the best solution among poor solutions.” The model can be used not only for ranking and selection, but even for overall performance gaps. As a result, the model provides a more accurate picture of the gap between the sustainability performance of pharmaceutical companies and their targets. This information helps stakeholders understand how a company is performing in terms of sustainability across all dimensions and indicators from target level to tolerable level. Based on the data obtained for AK Company, a Chinese pharmaceutical company, the empirical results show that there is considerable scope for optimizing the sustainability performance of AK Company. In particular, the dimension “Customer Perspective” and the indicator “customer relation” are the largest short board in the AK Company’s sustainability performance, which means that more needs to be done in terms of sustainability.

There are, of course, some limitations to this paper. Firstly, different evaluation systems are applicable to different companies and stakeholders, and they need to choose the most appropriate indicator system for their different needs. Therefore, specific evaluation indicators can be adjusted and optimized under the framework of this paper’s indicator system in the process of sustainability performance assessment. Secondly, the data obtained from the questionnaire is somewhat subjective. Despite having a good understanding of the actual situation of the case pharmaceutical company, it is impossible to avoid bias in the scoring of impact weights and satisfaction scores. Therefore, the effect of bias due to subjectivity was minimized by increasing the number of questionnaires distributed and the quality of questionnaire results in subsequent studies. Thirdly, the case study results about a Chinese pharmaceutical company may have some limitations in their generality and validity. Therefore, the model can be further applied and compared with data from pharmaceutical companies in different regions and countries. Therefore, the model can be further applied to assess the sustainable performance of pharmaceutical companies in different economic and regional contexts in a targeted manner.

## Data Availability

The original contributions presented in the study are included in the article/supplementary material, further inquiries can be directed to the corresponding author.

## References

[1] Rudnicka E Napierała P Podfigurna A Męczekalski B Smolarczyk R Grymowicz M (2020). The World Health Organization (WHO) approach to healthy ageing. Maturitas 139:6–11. doi: 10.1016/j.maturitas.2020.05.018, PMID: 32747042 PMC7250103

[2] Jung H Jeon J Choi H (2021). Important factors in the development of biopharmaceutical logistics centers. Asian J Shipp Logist 37:301–6. doi: 10.1016/j.ajsl.2021.07.003

[3] Shacham M Greenblatt-Kimron L Hamama-Raz Y Martin LR Peleg O Ben-Ezra M (2021). Increased COVID-19 vaccination hesitancy and health awareness amid COVID-19 vaccinations programs in Israel. Int J Environ Res Public Health 18:3804. doi: 10.3390/ijerph18073804, PMID: 33917327 PMC8038659

[4] Lim SH (2021). Promissory shock, broken future: COVID-19 and state-led speculations in biotechnology and pharmaceutical industries in South Korea. Appl Geogr 136:102560. doi: 10.1016/j.apgeog.2021.102560, PMID: 36536775 PMC9751681

[5] Maniar MS Kumar A Mentzer RA (2020). Global process safety incidents in the pharmaceutical industry. J Loss Prevent Proc 68:104279. doi: 10.1016/j.jlp.2020.104279, PMID: 39711777

[6] Belkhir L Elmeligi A (2019). Carbon footprint of the global pharmaceutical industry and relative impact of its major players. J Clean Prod 214:185–94. doi: 10.1016/j.jclepro.2018.11.204

[7] Enick OV Moore MM (2007). Assessing the assessments: pharmaceuticals in the environment. Environ Impact Assess Rev 27:707–29. doi: 10.1016/j.eiar.2007.01.001

[8] Meena VD Dotaniya ML Saha JK Patra AK (2015). Antibiotics and antibiotic resistant bacteria in wastewater: impact on environment, soil microbial activity and human health. Afr J Microbiol Res 9:965–78. doi: 10.5897/AJMR2015.7195, PMID: 38147025

[9] Krammer F (2020). SARS-CoV-2 vaccines in development. Nature 586:516–27. doi: 10.1038/s41586-020-2798-3, PMID: 32967006

[10] Vyas N Joshi A Malviya S Kharia A (2020). Reduced pharma supply chain in COVID-19: measures to reduce India’s reliance for active pharmaceutical ingredients on China and other countries. Indian J Pharm Educ Res 54:835–42. doi: 10.5530/ijper.54.4.175

[11] Mihaiu DM Șerban R-A Opreana A Țichindelean M Bratian V Barbu L (2021). The impact of mergers and acquisitions and sustainability on company performance in the pharmaceutical sector. Sustain For 13:6525. doi: 10.3390/su13126525

[12] Smith NC (2003). Corporate social responsibility: whether or how? Calif Manag Rev 45:52–76. doi: 10.2307/41166188

[13] Chabowski BR Mena JA Gonzalez-Padron TL (2011). The structure of sustainability research in marketing, 1958-2008: a basis for future research opportunities. J Acad Market Sci 39:55–70. doi: 10.1007/s11747-010-0212-7

[14] Shnayder L van Rijnsoever FJ Hekkert MP (2016). Motivations for corporate social responsibility in the packaged food industry: an institutional and stakeholder management perspective. J Clean Prod 122:212–27. doi: 10.1016/j.jclepro.2016.02.030

[15] Milanesi M Runfola A Guercini S (2020). Pharmaceutical industry riding the wave of sustainability: review and opportunities for future research. J Clean Prod 261:121204. doi: 10.1016/j.jclepro.2020.121204

[16] Aboud A Yang X (2022). Corporate governance and corporate social responsibility: new evidence from China. Int J Account Inf Manag 30:211–29. doi: 10.1108/IJAIM-09-2021-0195

[17] Castillo-Merino D Rodríguez-Pérez G (2021). The effects of legal origin and corporate governance on financial firms’ sustainability performance. Sustain For 13:8233. doi: 10.3390/su13158233

[18] Taghizadeh-Hesary F Zakari A Alvarado R Tawiah V (2022). The green bond market and its use for energy efficiency finance in Africa. China Financ Rev Int 12:241–60. doi: 10.1108/CFRI-12-2021-0225

[19] Kim HK Lee CW (2021). Relationships among healthcare digitalization, social capital, and supply chain performance in the healthcare manufacturing industry. Int J Environ Res Public Health 18:1417. doi: 10.3390/ijerph18041417, PMID: 33546393 PMC7913591

[20] Saki P Ebrahimnejad S (2015). An integrated approach for measuring the performance of suppliers in the pharmaceutical industry: a case study. Int J Logist Syst Manag 22:267–95. doi: 10.1504/IJLSM.2015.072283, PMID: 35009967

[21] SubbaNarasimha PN Ahmad S Mallya SN (2003). Technological knowledge and firm performance of pharmaceutical firms. J Intellect Cap 4:20–33. doi: 10.1108/14691930310455360

[22] Epstein MJ Wisner PS (2001). Using a balanced scorecard to implement sustainability. Environ Qual Manag 11:1–10. doi: 10.1002/tqem.1300

[23] Schaltegger S Wagner M (2006). Integrative management of sustainability performance, measurement and reporting. Int J Account Audit Perform Evaluation 3:1–19. doi: 10.1504/IJAAPE.2006.010098

[24] Albertini E (2013). Does environmental management improve financial performance? A meta-analytical review. Organ Environ 26:431–57. doi: 10.1177/1086026613510301

[25] Moslemi S Mirzazadeh A Weber GW Sobhanallahi AM (2021). Integration of neural network and AP-NDEA model for performance evaluation of sustainable pharmaceutical supply chain. Opsearch 59:1116–57. doi: 10.1007/s12597-021-00561-1, PMID: 39711969

[26] Bhattacharyya S Chatterjee S (2020). Efficiency evaluation of selected Indian pharmacceutical firms since inception of product patent. Int J Manag Concep Philos, 13:292–318. doi: 10.1504/IJMCP.2020.112196

[27] Tavana M Khalili-Damghani K Rahmatian R (2015). A hybrid fuzzy MCDM method for measuring the performance of publicly held pharmaceutical companies. Ann Oper Res 226:589–621. doi: 10.1007/s10479-014-1738-8

[28] Opricovic S Tzeng GH (2004). Compromise solution by MCDM methods: a comparative analysis of VIKOR and TOPSIS. Eur J Oper Res 156:445–55. doi: 10.1016/S0377-2217(03)00020-1

[29] Peng KH Tzeng GH (2017). Exploring heritage tourism performance improvement for making sustainable development strategies using the hybrid modified MADM model. Curr Issues Tourism 22:921–47. doi: 10.1080/13683500.2017.1306030, PMID: 39699680

[30] Shen KY Hu SK Tzeng GH (2017). Financial modeling and improvement planning for the life insurance industry by using a rough knowledge based hybrid MCDM model. Inf Sci 375:296–313. doi: 10.1016/j.ins.2016.09.055

[31] Hu KH Chen FH We WJ (2016). Exploring the key risk factors for application of cloud computing in auditing. Entropy 18:401. doi: 10.3390/e18080401

[32] Tzeng GH Shen KY. New concepts and trends of hybrid multiple criteria decision making. 1st ed. Boca Raton, FL: CRC Press/Taylor and Francis Group (2017).

[33] Doerr J. Measure what matters: How Google, bono, and the gates foundation rock the world with OKRs. 2nd ed. Portfolio/Penguin: New York, NY (2018).

[34] Niven PR Lamorte B. Objectives and key results: Driving focus, alignment, and engagement with OKRs. 1st ed. Wiley: Hoboken, NJ (2016).

[35] Jensen M (2001). Value maximisation, stakeholder theory, and the corporate objective function. Eur Financial Manag 7:297–317. doi: 10.1111/1468-036X.00158

[36] Kaplan RS Norton DP (1993). Putting the balanced scorecard to work. Harv Bus Rev 71:71–9. doi: 10.1016/B978-0-7506-7009-8.50023-9, PMID: 10119714

[37] Tsamenyi M Onumah J Tetteh-Kumah E (2010). Post-privatization performance and organizational changes: case studies from Ghana. Crit Perspect Account 21:428–42. doi: 10.1016/j.cpa.2008.01.002

[38] Silva S Nuzum A Schaltegger S (2019). Stakeholder expectationson sustainability performance measurement and assessment: A systematic literature review. J Clean Prod 217:204–15. doi: 10.1016/j.jclepro.2019.01.203

[39] Schaltegger S (2011). Sustainability as a driver for corporate economic success consequences for the development of sustainability management control. Soc Econ 33:15–28. doi: 10.1556/socec.33.2011.1.4, PMID: 29951292

[40] Figge F Hahn T Schaltegger S Wagner M (2002). The sustainability balanced scorecard-linking sustainability management to business strategy. Bus Strategy Environ 11:269–84. doi: 10.1002/bse.339

[41] Sundin H Granlund M Brown DA (2010). Balancing multiple competing objectives with a balanced scorecard. Eur Account Rev 19:203–46. doi: 10.1080/09638180903118736

[42] Voelpel SC Leibold M Eckhoff RA (2006). The tyranny of the balanced scorecard in the innovation economy. J Intellect Cap 7:43–60. doi: 10.1108/14691930610639769

[43] Griggs D Stafford-Smith M Gaffney O Rockström J Öhman MC Shyamsundar P (2013). Sustainable development goals for people and planet. Nature 495:305–7. doi: 10.1038/495305a, PMID: 23518546

[44] Knoepfel I (2001). Dow Jones sustainability group index: a global benchmark for corporate sustainability. Corp Environ Strategy 8:6–15. doi: 10.1016/S1066-7938(00)00089-0

[45] Esteban D (2008). Strengthening corporate social responsibility in the pharmaceutical industry. J Medical Mark 8:77–9. doi: 10.1057/palgrave.jmm.5050126

[46] Schneider JL Wilson A Rosenbeck JM (2010). Pharmaceutical companies and sustainability: an analysis of corporate reporting. Benchmarking An Int J 17:421–34. doi: 10.1108/14635771011049371

[47] Joshi R Kumar S (2019). An intuitionistic fuzzy information measure of order-(α,) with a new approach in supplier selection problems using an extended VIKOR method. J Appl Math Comput 60:27–50. doi: 10.1007/s12190-018-1202-z

[48] Searcy C (2012). Corporate sustainability performance measurement systems: A review and research agenda. J Bus Ethics 107:239–53. doi: 10.1007/s10551-011-1038-z

[49] Dkhili H (2023). Does environmental, social and governance (ESG) affect market performance? The moderating role of competitive advantage. Compet Rev Int Bus J. 34:327–352. doi: 10.1108/CR-10-2022-0149

[50] Xu J Liu F Shang Y (2020). R&D investment, ESG performance and green innovation performance: Evidence from China. Kybernetes 50:737–56. doi: 10.1108/K-12-2019-0793, PMID: 35579975

[51] Speckbacher G Bischof J Pfeiffer T (2003). A descriptive analysis on the implementation of balanced scorecards in German-speaking countries. Manag Account Res 14:361–88. doi: 10.1016/j.mar.2003.10.001

[52] Orlitzky M Swanson DL (2012). Assessing stakeholder satisfaction: toward a supplemental measure of corporate social performance as reputation. Corp Reput Rev 15:119–37. doi: 10.1057/crr.2012.3

[53] Psomas EL Fotopoulos CV Kafetzopoulos DP (2011). Motives, difficulties and benefits in implementing the ISO 14001 environmental management system. Manag Environ Qual 22:502–21. doi: 10.1108/14777831111136090

[54] George-Ufot G Qu Y Orji IJ (2017). Sustainable lifestyle factors influencing industries’ electric consumption patterns using fuzzy logic and DEMATEL: the Nigerian perspective. J Clean Prod 162:624–34. doi: 10.1016/j.jclepro.2017.05.188

[55] Hsu CW Kuo TC Chen SH Hu AH (2013). Using DEMATEL to develop a carbon management model of supplier selection in green supply chain management. J Clean Prod 56:164–72. doi: 10.1016/j.jclepro.2011.09.012

[56] Wu KJ Hou W Wang Q Yu R Tseng ML (2022). Assessing city’s performance-resource improvement in China: A sustainable circular economy framework approach. Environ Impact Assess Rev 96:106833. doi: 10.1016/j.eiar.2022.106833

[57] Hair JF Howard MC Nitzl C (2020). Assessing measurement model quality in PLS-SEM using confirmatory composite analysis. J Bus Res 109:101–10. doi: 10.1016/j.jbusres.2019.11.069

[58] Cepeda-Carri G Henseler J Ringle CM Rold’an JL (2016). Prediction-oriented modeling in business research by means of PLS path modeling: introduction to a JBR special section. J Bus Res 69:4545–51. doi: 10.1016/j.jbusres.2016.03.048, PMID: 39711777

[59] Robert S Kaplan DP Norton (2001). Transforming the balanced scorecard from performance measurement to strategic management: part I. Account Horiz 15:147–60. doi: 10.2308/acch.2001.15.2.147

[60] Horváth Partners. Balanced scorecard v praxi, vol. xiv. Praha: Profess Consulting (2012).

[61] Garg S Bhardwaj R (2024). Exploring the influence of factors causing stress among doctoral students by combining fuzzy DEMATEL-ANP with a triangular approach. Scientometrics 129:4695–719. doi: 10.1007/s11192-024-05108-x, PMID: 39711969

[62] Kao-Yi S Min-Ren Y Gwo-Hshiung T (2014). Combining VIKOR-DANP model for glamor stock selection and stock performance improvement. Knowl Based Syst 58:86–97. doi: 10.1016/j.knosys.2013.07.023, PMID: 39711777

[63] Yixiong F Zhaoxi H Guangdong T Zhiwu L Jianrong T Hesuan H (2018). Environmentally friendly MCDM of reliability-based product optimisation combining DEMATEL-based ANP, interval uncertainty and Vlse Kriterijumska Optimizacija Kompromisno Resenje (VIKOR). Inf Sci 442-443:128–44. doi: 10.1016/j.ins.2018.02.038, PMID: 39711777

[64] Feldmann FG Birkel H Hartmann E (2022). Exploring barriers towards modular construction- A developer perspective using fuzzy DEMATEL. J Clean Prod 367:133023. doi: 10.1016/j.jclepro.2022.133023

[65] Peng KH Tzeng GH (2013). A hybrid dynamic MADM model for problems-improvement in economics and business. Technol Econ Dev Econ 19:638–60. doi: 10.3846/20294913.2013.837114

[66] Tian G Liu X Zhang M Yang Y Zhang H Lin Y (2019). Selection of take-back pattern of vehicle reverse logistics in China via Grey-DEMATEL and fuzzy-VIKOR combined method. J Clean Prod 220:1088–100. doi: 10.1016/j.jclepro.2019.01.086

[67] Chen FH Tzeng GH (2015). Probing organization performance using a new hybrid dynamic MCDM method based on the balanced scorecard approach. J Test Eval 43:924–37. doi: 10.1520/JTE20130181

[68] Chen Z Ming X Zhang X Yin D Sun Z (2019). A rough-fuzzy DEMATEL-ANP method for evaluating sustainable value requirement of product service system. J Clean Prod 228:485–508. doi: 10.1016/j.jclepro.2019.04.145

[69] Hu KH Chen FH Tzeng GH Lee JD (2015). Improving corporate governance effects on an enterprise crisis based on a new hybrid DEMATEL with the MADM model. J Test Eval 43:1395–412. doi: 10.1520/JTE20140094

[70] Mavi RK Standing C (2018). Critical success factors of sustainable project management in construction: A fuzzy DEMATEL-ANP approach. J Clean Prod 194:751–65. doi: 10.1016/j.jclepro.2018.05.120

[71] Roy M Sen P Pal P (2020). An integrated green management model to improve environmental performance of textile industry towards sustainability. J Clean Prod 271:122656. doi: 10.1016/j.jclepro.2020.122656

[72] Wu HY Lin YK Chang CH (2011). Performance evaluation of extension education centers in universities based on the balanced scorecard. J Eval Program Plann 34:37–50. doi: 10.1016/j.evalprogplan.2010.06.001, PMID: 20619892

[73] Mani V Agrawal R Sharma V Kavitha TN (2016). Socially sustainable business practices in Indian manufacturing industries: a study of two companies. Int. J. Logist. Syst. Manag. 24:18–44. doi: 10.1504/IJLSM.2016.075661, PMID: 35009967

[74] Sahu K Kohli S (2019). Performance improvement tool towards the medicines manufacturing pharmaceutical companies under sustainable practices. Int J E-entrepreneursh Innov 9:35–48. doi: 10.4018/IJEEI.2019070103

[75] Chaar BB Lee J (2012). Role of socioeconomic status on consumers’ attitudes towards DTCA of prescription medicines in Australia. J Bus Ethics 105:447–60. doi: 10.1007/s10551-011-0977-8

[76] Creamer G Freund Y (2010). Learning a board balanced scorecard to improve corporate performance. Decis Support Syst 49:365–85. doi: 10.1016/j.dss.2010.04.004

[77] Eccles RG Grant R van Riel CBM (2006). Reputation and transparency: lessons from a painful period in public disclosure. Long Range Plan 39:353–9. doi: 10.1016/j.lrp.2006.09.004

[78] Maltz AC Shenhar AJ Reilly RR (2003). Beyond the balanced scorecard: refining the search for organizational success measures. Long Range Plan 36:187–204. doi: 10.1016/S0024-6301(02)00165-6

[79] Lorenzini GC Mostaghel R Hellström D (2018). Drivers of pharmaceutical packaging innovation: a customer-supplier relationship case study. J Bus Res 88:363–70. doi: 10.1016/j.jbusres.2017.11.030

[80] Veleva V Bodkin G Todorova S (2017). The need for better measurement and employee engagement to advance a circular economy: lessons from Biogen’s “zero waste” journey. J Clean Prod 154:517–29. doi: 10.1016/j.jclepro.2017.03.177

[81] Saeed BB Afsar B Hafeez S Khan I Tahir M Afridi MA (2019). Promoting employee’s proenvironmental behavior through green human resource management practices. Responsib Environ Manag 26:424–38. doi: 10.1002/csr.1694

[82] Bom S Jorge J Ribeiro HM Marto J (2019). A step forward on sustainability in the cosmetics industry: a review. J Clean Prod 225:270–90. doi: 10.1016/j.jclepro.2019.03.255

[83] Agrawal S Singh R. K. Murtaza Q. (2016). Outsourcing decisions in reverse logistics: Sustainable balanced scorecard and graph theocratic approach. Resour Conserv Recy, 108:41–53. doi: 10.1016/j.resconrec.2016.01.004

[84] Sudhakar MP Kumar BR Mathimani T Arunkumar K (2019). A review on bioenergy and bioactive compounds from microalgae and macroalgae-sustainable energy perspective. J Clean Prod 228:1320–33. doi: 10.1016/j.jclepro.2019.04.287

[85] Ángel del Brío J Junquera B Ordiz M (2008). Human resources in advanced environmental approaches--a case analysis. Int J Prod Res 46:6029–53. doi: 10.1080/00207540701352094

[86] Jabbour CJC (2013). Environmental training in organizations: from a literature review to a framework for future research. Resources Conserv Recycl 74:144–55. doi: 10.1016/j.resconrec.2012.12.017

[87] Zhu B-W Zhang J-R Tzeng G-H (2017). Public Open Space Development for Elderly People by Using the DANP-V Model to Establish Continuous Improvement Strategies towards a Sustainable and Healthy Aging Society. Sustainability, 9, 420. doi: 10.3390/su9030420

